# Information seeking and evaluation: a multi-institutional survey of veterinary students

**DOI:** 10.5195/jmla.2019.674

**Published:** 2019-10-01

**Authors:** Erin R. B. Eldermire, Suzanne Fricke, Kristine M. Alpi, Emma Davies, Andrea C. Kepsel, Hannah F. Norton

**Affiliations:** Head, Flower-Sprecher Veterinary Library, Cornell University Library, Veterinary College, Cornell University, Ithaca, NY, erb29@cornell.edu; Animal Health Sciences Librarian, Animal Health Library, Washington State University, Pullman, WA, suzanne.fricke@wsu.edu; University Librarian, OHSU Library, Oregon Health & Science University, Portland, OR, alpi@ohsu.edu; Associate Clinical Professor, Section of Neurology/Neurosurgery Section Chief, Cornell University, Ithaca, NY, ed445@cornell.edu; Health Sciences Educational Technology Librarian, University Libraries, Michigan State University, East Lansing, MI, akepsel@msu.edu; Health Science Center Libraries, University of Florida, Gainesville, FL, nortonh@ufl.edu

## Abstract

**Objective:**

To practice evidence-based medicine, clinicians must be competent in information literacy (IL). Few studies acknowledge the critical role that reading strategies play in IL instruction and assessment of health professional students. The purpose of this study was to understand the information-seeking and evaluation behaviors of doctor of veterinary medicine (DVM) students in regard to scientific papers.

**Methods:**

The authors studied DVM student behaviors across eight programs in North America using a web-based survey of closed- and open-ended questions about finding and evaluating scientific papers, including a task to read a linked scientific paper and answer questions about it.

**Results:**

A total of 226 individuals responded to the survey. The sections of a scientific paper that were most commonly read were the abstract, introduction, and conclusions. Students who reported reading a higher proportion of scientific papers were more likely to feel confident in their abilities to interpret them. A third of respondents answered open-ended questions after the paper reading task. Respondents felt the least amount of confidence with one of the final steps of evidence-based medicine, that of interpreting the significance of the paper to apply it in veterinary medicine.

**Conclusions:**

DVM students may lack the skills needed to evaluate scientific literature and need more practice and feedback in evaluating and interpreting scientific papers. Librarians who support DVM students can (1) help DVM students to efficiently evaluate scientific literature, (2) seek training opportunities in alternative modes of teaching and learning IL skills, and (3) partner with veterinary faculty and clinicians to provide students with practice and feedback in information evaluation.

## INTRODUCTION

Like all health professionals, veterinarians are encouraged to practice evidence-based medicine throughout their careers, which means they need to apply the best available evidence to support their decision making. Information literacy (IL) is defined as having the ability to “recognize when information is needed and have the ability to locate, evaluate, and use effectively the needed information” [1]. These are all skills that are essential for veterinarians to practice effective evidence-based medicine, and IL is an acknowledged essential skill for health professionals.

In North America, doctor of veterinary medicine (DVM) programs are four-year, post-secondary degrees composed of didactic, small group, and clinical components, making them comparable to most doctor of medicine and doctor of osteopathic medicine programs. The American Veterinary Medical Association (AVMA), which accredits DVM programs, requires each program to “demonstrate, using its outcomes assessment data, that students are competent in retrieving, evaluating and efficiently applying information through the use of electronic and other appropriate information technologies” [2].

IL skills and standards have been studied in health profession education [3–7], but little IL research in veterinary education has been reported. The few researchers generally concluded that DVM students need to develop stronger IL skills. Weiner et al., who reported on the information-seeking behaviors of first-year DVM students, found DVM students exercised some IL skills but that they could be strengthened [8]. A survey by Elnoor et al. found that many DVM students lacked basic IL skills, “which is a serious concern” [9]. A 2011 survey of veterinary librarians detailed deficiencies in students’ IL skills and librarian’s struggles to incorporate IL into the DVM curriculum [10]. In 2015, a survey of veterinary teaching faculty and librarians concluded that evidence-based medicine skills were not taught consistently, either within or across institutions [11].

Many IL models start with the assumption that students access information through reading, but this may not always be the case [12]. In addition, the skill of reading is not a new challenge that medical students face. In 1974, one study noted that “many medical students experienced academic difficulties which appeared to stem from deficiencies in reading skills” [13]. In a 2001 survey of academic deans of veterinary schools, thirteen of twenty-seven respondents noted that their students had a weakness in reading professional journals [14]. Since that study was published, not only is information presented in a broader variety of formats, but there is also a concern that electronically presented text encourages skimming, rather than deep reading [15]. Deep reading is associated with critical thinking, a skill taught traditionally through journal clubs [16–20]. However, newer approaches to critical thinking emphasize repeated problem-based practice in making evidence-based and data-informed decisions [21].

Today’s digital information landscape offers diverse avenues for gathering information beyond the print reading environment, thus information-seeking skills have become increasingly important. Studies suggest that IL skills instruction is most effective when integrated into the curriculum and when librarians are supported by clinical faculty [22, 23]. However, there is evidence that some students might forget IL skills they have learned [24]. This further supports the idea that teaching IL skills should be integrated throughout the curriculum over the entire four-year course of study.

The information-seeking habits and skills that students establish early in life may have implications for evidence-based practice in health care professions that still rely heavily on reading. Although there are many approaches to addressing IL skills, the authors elected to ask about reading skills and behaviors of veterinary student populations. Primarily, we surveyed students about where they look for information and focused on their ability to evaluate that information [25–28].

We acknowledge that this focus on reading may be an oversimplified approach in today’s information environment, as students engage with information through multiple modes, including listening, touch, observation, and action. A preliminary study found that DVM students’ confidence and ease in reading scientific literature did not differ between cohorts, but third-year students spent more time reading scientific papers than first- and second-year students [28]. Absent other studies examining DVM students’ reading behaviors and motivations, we consider that students are known to place greater importance on information that is both assessed for comprehension and has clinical application [29]. Reading and making sense of scientific literature is an important piece of the larger entrustable professional activity, “Formulate relevant questions and retrieve evidence to advance care” [30].

This study of DVM students across eight programs in North America explores their information-seeking behaviors and attitudes toward scientific literature, as well as their confidence in and perceptions of evaluating this literature. The objective of this study was to understand DVM students’ information-seeking and evaluation behaviors in regard to scientific literature. To address their information-seeking behaviors, we asked why and how DVM students access scientific literature. To address their evaluation behaviors, we asked (1) what strategies students used to read and evaluate scientific literature, (2) how easy or difficult students found reading scientific literature, (3) how confident students were in interpreting scientific literature, and (4) whether there was a relationship between ease and confidence when DVM students read scientific literature.

## METHODS

A survey was developed in Qualtrics [31] by librarian and veterinary instructor researchers at Cornell University [28, 32]. A survey prototype was shared with three DVM students, and their feedback was incorporated. The survey comprised two parts. Part 1 included eighteen questions that were either multiple choice, Likert-scale, or open-ended and asked general demographic information as well as frequency, motivations, practices, and perceptions of reading scientific papers. Part 2 asked students to read an open access scientific paper that was linked in the survey [33], after which they were asked twelve questions about sections of the paper that they read, the amount of time that they took to read the paper, and conclusions that they drew from reading the paper.

In June 2016, the first author contacted librarians from veterinary schools to explore using this two-part survey as the basis of a multi-institutional study to investigate student perceptions around scientific information literacy. Librarians from twenty-eight institutions initially expressed interest. Fifteen of those institutions participated in early planning discussions about timing, role of the librarians in the study, changes to survey questions, promotional strategies, and the use of Qualtrics software for survey distribution. Requirements for institutional review board (IRB) approval were also addressed. Several changes were made to the survey based on the recommendations of participating librarians, varying from simplifying the method of survey distribution to modifying specific questions to make them meaningful across institutions. The full survey instrument appears as a supplemental appendix.

### Survey distribution and data collection

Eight DVM programs in the United States and Canada elected to distribute the survey: seven submitted the research for IRB review at their own institutions, and one facilitated the distribution of the survey under the original institution’s IRB exemption. The IRB process and project information that was required varied by participating institution. The mean time from submission to exemption was ten days (range of two to thirty-one days).

The initial plan was to administer the survey confidentially from the first author’s Qualtrics account, with the ability to send targeted reminders to nonresponders. We determined that it was not possible to do so without providing student emails to the primary administering institution (Cornell University). Therefore, several institutions administered the survey through an anonymous link, enabling it to be disseminated to student class email lists through the dean’s office or directly by the authors. Participation was voluntary, and those who started the survey were not required to finish. Survey respondents included in this study were first-, second-, third-, and fourth-year DVM students across multiple institutions. Participating DVM programs represented 7 public land-grant universities and 1 private university. Class sizes ranged from 60–130 individuals per year.

As participating institutions did not have synchronized academic calendars, the survey distribution was staggered based on IRB approval dates and varying institutional processes. Data were collected September to November 2016. At the institutions that allowed it, a reminder was sent two weeks after the initial distribution. The first author extracted the data in Qualtrics for all sites and, to ensure anonymity, checked open-ended responses to redact any identifiable information as well as to confirm that no demographic categories had fewer than five responses.

### Data analysis

Quantitative and qualitative data were collected concurrently, analyzed separately, and integrated to inform the final discussion [34]. For quantitative analysis, descriptive statistics were prepared using Excel. Within-subjects comparisons were made using paired *t*-tests, and we visually examined the data using scatter plots before running correlations between variables. Correlations between responses to survey questions were performed using SPSS statistical software (version 25).

For qualitative analysis, two separate analysts applied basic interpretive and constant comparative methods to the open-ended survey responses. First, we used basic interpretive methods [35] of data reduction to code for and capture themes of facilitating and hindering elements. Because two investigators coded, we compared applied codes and re-coded again using the constant comparative method [36]. Participants’ words were retained as the labels for the coding variables and themes where possible. The combined code listing was then applied to see if any of the responses offered insights that were not already covered by the previously created themes. The final analysis consisted of a list of agreed upon themes with exemplar quotes.

## RESULTS

Of the 3,501 students who received an invitation to the survey, 226 participated, for an overall response rate of 6.5%. Of these, 157 (69.5%) continued to part 2 of the survey and responded to at least 1 question after reading the linked paper. Seventy-six students (33.6%) responded to 1 or more open-ended questions after reading the paper. Response rates by institution ranged from 2.1% to 14.3%. Respondents were 81.9% female and 17.7% male (1 individual preferred not to answer about their gender); this distribution reflected DVM student gender demographics in 2016 [37].

In terms of their progression through the DVM curriculum, 27.9% were in their first year, 27.0% second year, 28.8% third year, and 15.9% fourth year, with 1 individual in a DVM/PhD program. Students could select multiple options for their academic experience prior to attending veterinary school. Almost all (91.6%) had a bachelor’s degree, 5.3% some college or university but no bachelor’s degree, 8.4% a master’s degree, 2.2% a professional degree (e.g., law, medicine, etc.), 0.4% a PhD, and 2.2% other academic experience. A larger percentage of students with higher degrees (professional, master’s, or PhD) responded to our survey (11.1%) than existed in the DVM student population as a whole (5.2%; master’s and PhD) [38].

**Figure 1:**
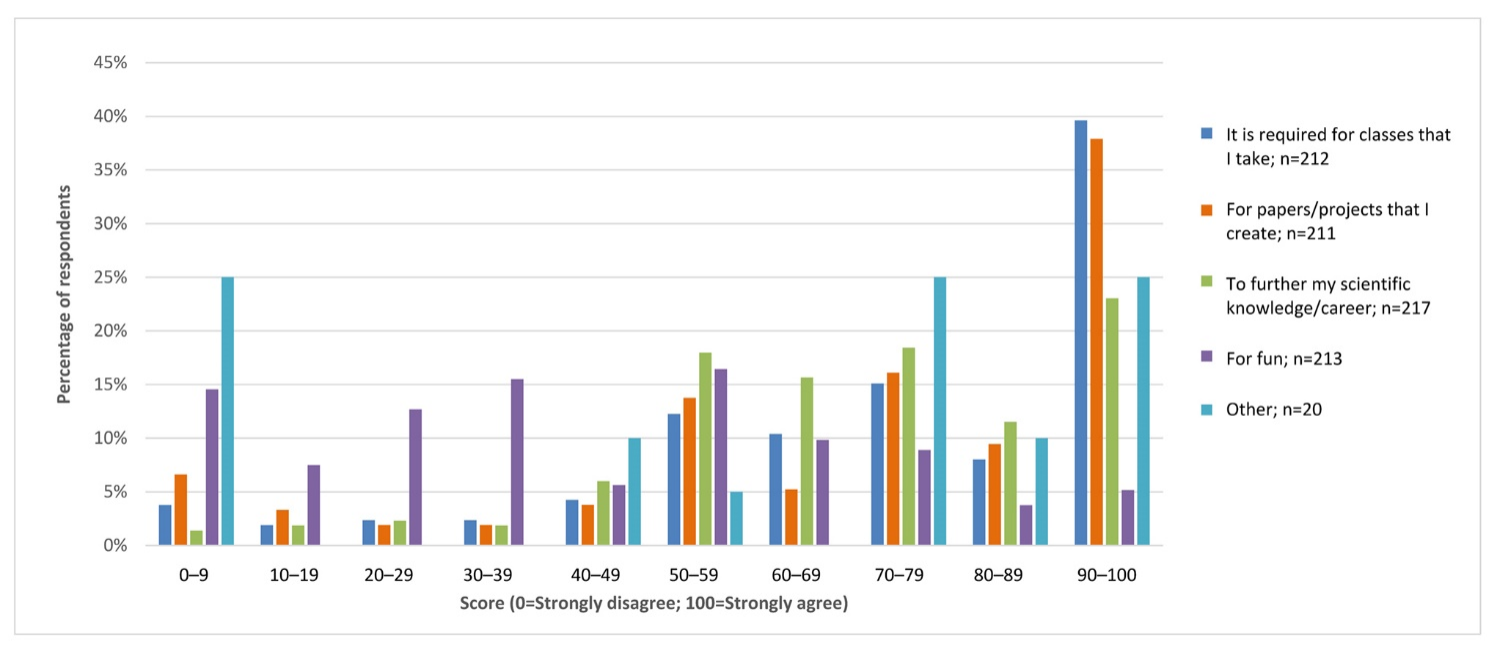
Reasons why veterinary students read scientific papers

To address why DVM students accessed the scientific literature, they were asked the extent to which they agreed with statements about why they read scientific papers, on a scale of 0 (Strongly disagree) to 100 (Strongly agree) (Q8–Q12). Their level of agreement differed significantly across options (n=225, ANOVA, F(4,852)=42.5, *p*<0.001) with the following average ratings: “because it is required for the classes I take” (73.2), “for papers/projects that I create” (71.0), “to further my scientific knowledge/career” (69.0), and “for fun” (45.6). Open text responses for “Other” (58.9) included to fact check or learn more about science news stories, to look up evidence on new treatment protocols, to use for work, to supplement class material, and to look up current information on a specific subject. Distribution of responses in deciles are shown in Figure 1.

For the question about how students discover scientific papers that they read (Q13), the most popular responses were searching a specific database, browsing specific journals, and getting recommendations from friends or colleagues. Full results are shown in Table 1.

**Table 1:** How do you discover scientific papers that you read? (Q13; n=218)

	# Responses	% Responses
I search a specific database (e.g., PubMed, Google Scholar) or website (e.g., journal website, library website)	188	86.2%
I browse specific journals	100	45.9%
I get recommendations from friends or colleagues	82	37.6%
From Blackboard, Moodle, Classlist, or similar	70	32.1%
I find it via Facebook, Twitter, ResearchGate, or other social media sites	68	31.2%
I read what is recommended for my journal club	24	11.0%
I subscribe to table of contents alerts	18	8.3%
Other	13	6.0%

The questions also addressed what strategies DVM students used to read scientific literature after its discovery and access. Students were asked, in general, what proportion of a scientific paper they read, on a scale of 0–100% (Q7). Their responses were grouped as follows: 33.9% reported reading 75%–100% of a paper, 38.0% reported reading 50%–75%, 18.6% reported reading 25%–49%, and 9.5% reported less than 25%. To further understand their reading strategies, students who completed the paper-reading task were asked, using survey logic, a series of questions about which sections of the paper they read and whether this reflected how they generally read scientific papers (Q20–23). The most common sections typically read included: abstract, title, conclusions, and introduction/background. Full responses to this series of questions are shown in Table 2.

**Table 2:** What sections of the paper do you typically read? (Q23; n=150)

	# Responses	% Responses
Abstract	144	96.0%
Title	138	92.0%
Conclusions	129	86.0%
Introduction/Background	118	78.7%
Results	101	67.3%
Discussion	101	67.3%
Methods	72	48.0%
Authors and author affiliations	69	46.0%
References	32	21.3%
Additional material	31	20.7%
Acknowledgments	29	19.3%

**Figure 2:**
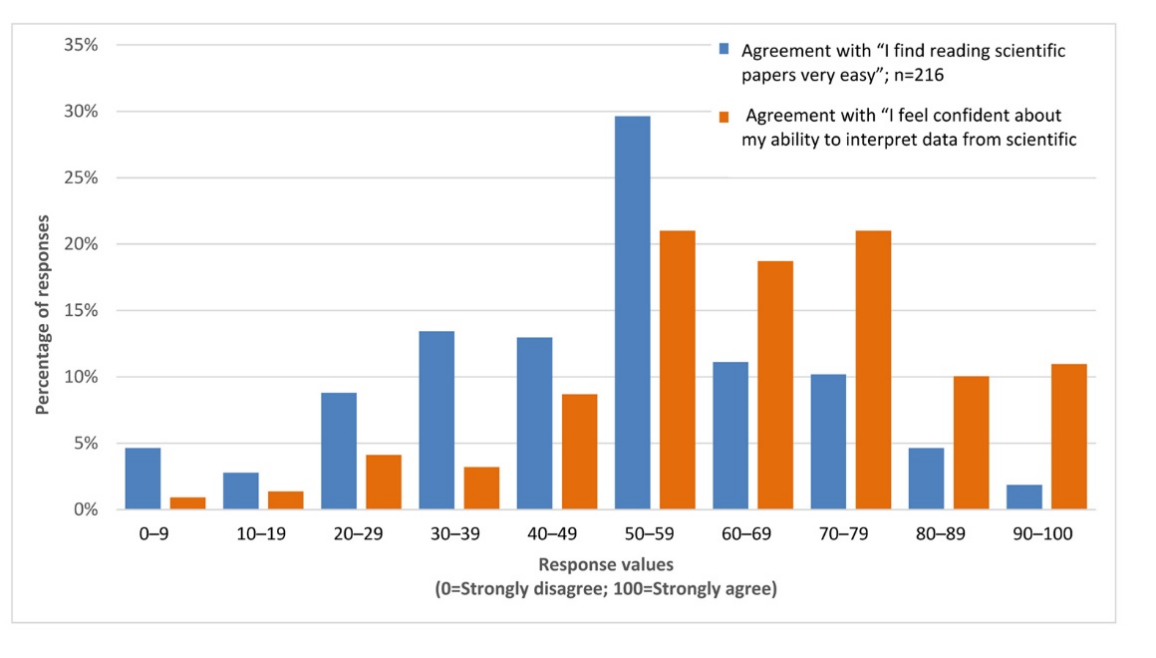
Ease in reading scientific papers and confidence in interpreting papers’ data

Two questions focused on students’ perceptions of the ease of reading scientific papers and their confidence in interpreting the results, each with a following open-ended question, “Why?” First, the extent to which they agreed with the statement, “I find reading scientific papers very easy,” on a scale of 0 (Strongly disagree) to 100 (Strongly agree) was assessed (Q15). The mean was 48.5, with distribution of responses in deciles shown in Figure 2. Second, students were asked the extent to which they agreed with the statement “I feel confident about my ability to interpret data from scientific papers,” on a scale of 0 (Strongly disagree) to 100 (Strongly agree) (Q17). The mean was 64.1, with distribution of responses in deciles shown in Figure 2.

Students who read the embedded paper were also asked about their confidence in understanding the contents of that specific paper (Q25). They were significantly less confident in their ability to interpret that paper (n=119, mean 55.4, paired *t*-test, t(235)=2.96, *p<*0.001), compared to their overall confidence in their ability to interpret scientific papers (Q17). Students’ ease in reading scientific papers (Q15) and overall confidence in interpreting them (Q17) were not correlated (n=210, R^2^= −0.01, *p*=0.99).

A priori, we knew many of the survey questions were related, so we investigated potential relationships between them by running linear regression analysis and analysis of variance (ANOVA) on ease of reading scientific papers, confidence in interpreting scientific papers, love of reading scientific papers, proportion of papers read, reasons scientific papers are read, sections of papers read, and demographic variables.

Several small but statistically significant correlations were found. Students who agreed more strongly with the statement, “I love to read scientific papers” (Q14), were likely to agree more strongly with the statement, “I feel confident about my ability to interpret data from scientific papers” (Q17) (n=211, R^2^=0.16, *p<*0.001). Students who reported reading a higher proportion of scientific papers (Q7) were more likely to feel confident in their ability to interpret them (Q17) (n=214, R^2^ 0.05, *p<*0.001). The more sections of a paper that students read in the paper reading task (Q20 and Q21), the greater confidence they had in interpreting scientific papers (Q17) (n=150, R^2^ 0.05, *p=*0.01).

### Thematic analysis from open-ended questions

Four survey questions with numerical scores were followed by open-ended “Why?” questions (Q16, Q18, Q28, and Q30). We analyzed responses to two of these “Why?” questions in part 1, which followed the survey questions: (Q15) “I find reading scientific papers very easy” (Q16 “Why?”, n=128) and (Q17) “I feel confident about my ability to interpret data from scientific papers” (Q18 “Why?”, n=95). Responses to why participants found reading easy or were confident in reading were largely similar. Four respondents to the second question referred back to their answers to the first question.

The themes coded from responses to these 2 questions appear in Table 3. They were categorized into attributes of the student, the work, and the reading event, as well as student strategies for learning. Of the 226 respondents, 76 (33.6%) answered at least 1 open-ended question about the embedded paper. However, we did not analyze responses to Q28 and Q30, because response rates to the questions were low and most pertained to the topic of the paper (metacognition) and not evaluation of the paper. Thus, the thematic analysis only applied to respondents’ general sentiments as expressed in answers to Q16 and Q18. Chi-squared analysis indicated that 2 of the demographic characteristics of those responding to open-ended questions were not significantly different than those of the full respondent pool (gender, χ^2^(2)=1.353, *p*=0.51; previous academic experience, χ^2^(4)=3.827, *p*=0.43) and 1 was significantly different (year in DVM program, χ^2^(4)=10.6, p=0.03). Fourth-year students were over-represented among open-ended responses, while first- and third-year students were slightly underrepresented.

**Table 3:** Themes for responses to open-ended questions (Q16 and Q18)

Domain of theme	Researcher-generated categorization	In the students’ words
Ease and confidence in reading scientific papers is based on…	Self-identified characteristics of the student	Familiarity with topic Interest in topic Experience of student through education and training Experience of student by practicing
	Characteristics of the paper	Type of study Focus/audience/level of reading Statistics Terminology/acronyms How well written How organized
	Characteristics of the reading event	Time to find, read, and evaluate a paper in context of other works Information need/assignment vs. patient need vs. professional development Amount of interpretation required
Strategies for learning:	Sections of the paper read	
	Recognition of need for additional reading	
	Preference for learning through experience or from a mentor	

The most common theme involved the positive effect of prior experience and/or interest on a student’s confidence and ease in reading, which included codes such as practice with critical reading in a variety of courses or settings as well as interest or background in the topic, and the negative impact of lack of interest in or unfamiliarity with the topic. One respondent noted:

It can be challenging, mainly because I’m not an expert on the topics I’m reading about and the articles often go very in depth. Also because I’m still learning how to critically evaluate research and recognize flaws in experiment design or conclusions.

Students at all levels of experience recognized the difficulty of reading and interpreting scientific literature. Lack of academic preparation in statistics and methodology was very frequently mentioned, and only the scale of it differed by respondents; for example, those lacking basic statistics commented generally while those with more statistical experience commented that more advanced statistical techniques were beyond them. One survey respondent noted, “I don’t understand some of the statistical methods talked about, so I usually skip that part.” Another said, “Looking at the data is one thing but actually understanding its implication is often difficult unless spelled out for me in the discussion.” These emerged as voiced struggles with those aspects of evidence-based medicine most frequently associated with critical appraisal.

Characteristics of the paper mattered a great deal, especially when the subject matter was difficult and the paper was dense, hard to understand, and written for experts. Several respondents commented on how the quality of writing and a clear format made the work easier to understand and more accessible. A very common problem complicating reading scientific papers was jargon or unfamiliar terminology or research techniques. The challenges of evaluating literature could come from other angles, as indicated by this comment:

I’m usually pretty good [at] figuring out what they were trying to convey to the reader. What their purpose was. When asked to figure out what inaccuracies or what could have rendered the paper biased I struggle.

Motivations for, and characteristics of, a reading event appeared in comments much less frequently. Although an earlier question prompted students to respond about whether they read for themselves or to complete an assignment, a few spontaneously added in these open-ended responses that they sought information for patient care. Time was the main factor impacting reading behaviors. One student commented, “the biggest reason why I dislike the scientific literature—having to wade through numerous papers to find the information I’m looking for.” Finally, students identified the least amount of confidence with one of the final steps of evidence-based medicine, that of interpreting the significance of reading for application in veterinary medicine.

Students identified several strategies for information gathering. When reading a paper, they skipped complicated parts and usually relied on the abstract or discussion. To be efficient, some looked only in particular subsections for specific information, but a few critiqued more deeply or worked harder to reread or look up unfamiliar language. Some mentioned the need to consult other sources and to “place the paper within a larger scientific context.” Finally, a few stated a preference for learning through experience and the advice of mentors.

## DISCUSSION

This study provides baseline information on the information-seeking and information-evaluation behaviors of DVM students across various curricula in North America. Our results suggest that DVM students primarily read scientific papers for course- or project-driven needs or to further scientific knowledge and feel that they receive the least amount of practice and feedback in evaluating and interpreting significance.

Reading scientific papers is a learned skill and learning how to use the structure of scientific papers is essential for efficiency [39]. Our findings that most respondents read the title, abstract, introduction/background, and conclusions, and that fewer read the methods, results, and discussion was consistent with Nielsen et al., who found that 85% of practicing veterinarians in the United Kingdom who responded to their survey read the abstract and conclusions, and only 29% read the materials and methods sections [40].

One way that librarians can address this selective reading for efficiency’s sake in teaching to search for and select relevant evidence is to have students explicitly start their evaluations with the methodology and results shown in the abstract because that was a component that almost all participants reported reading. This should raise questions and reveal possible gaps that can be made explicit so that students can go into a paper with that in mind. They can then check on their initial assessment by looking at certain elements in the methods and results sections to ensure that they are consistent or further explain the questions that arise from reading the abstract alone. Encouraging them to save their time by only reading the last paragraph of the introduction to confirm the purpose of the study and how it relates to their questions is a strategy to recover time that can be used for the methods or results. Trying to convince students to change their practice and read the methods section in its entirety earlier in the process might not be successful.

Students identified challenges with evaluating scientific articles, particularly in interpreting the validity of methodology and significance. This difficulty of reading and interpreting scientific literature was noted by many in responses that followed the statement, “I feel confident about my ability to interpret scientific papers.” As in other health professions, the lack of academic preparation in statistics and methodologies was a common lament [41, 42]. Although some respondents had formal training, it still might not be adequate, as one respondent commented:

I do not believe that the one class of statistics that I was required to take for veterinary school accurately prepared me to analyze the data of scientific papers.

Several noted a lack of access to feedback on their skills in interpreting scholarly papers, and this may be an area for future research, particularly how clinical rotations can use journal club activities in combination with repeated problem-based practice [21] to help students gain feedback on these skills. Another mechanism for training in evaluation is the development of critically appraised topics in veterinary medicine, which has been done in several veterinary programs in Europe [43] and as early as the first year of the DVM program at Mississippi State University [44].

Our findings support the notion that DVM students have difficulty reading scientific papers and that they attribute this to several factors. Respondents noted that some scientific papers included too much jargon, dense text, or unfamiliar terminology to be understandable. Greene encourages scientists to write papers with language that is more accessible and easily understood [45], and we suggest that scientists evaluate their writing with practitioners in mind to increase comprehension.

Other challenges that DVM students noted included low self-confidence in their evaluation skills, difficulty understanding statistics, lack of time, or inadequate training in reading scientific papers. Respondents reported seeing value in planning, feedback, and repetition to improve their skills. Several voiced concerns about lack of access to feedback that is needed to improve skills, including feedback on the correct interpretation of scholarly papers. Students who agreed more strongly with the statement, “I feel confident about my ability to interpret data from scientific papers,” were more likely to report reading a higher proportion of scientific papers. This suggests that practice in reading, evaluating, and interpreting scientific papers is associated with student confidence and deserves more attention across DVM curricula in North America if the goal is to produce veterinarians who are confident in using the scientific literature. The veterinary literature recognizes the need for practitioners to refresh their appraisal skills [46].

DVM students can come from a variety of backgrounds in which no standardized IL training has been established. Thus, we believe that the responsibility to teach all aspects of IL skills necessary for evidence-based practice lies with the accredited institutions who are training DVM students to become practitioners. We suggest purposefully integrating these skills into DVM learning targets in alignment with AVMA’s “Information Resources” standard for accreditation [2], which requires evaluation and other IL skills to be demonstrated through assessment.

Diverse instructional strategies are available and needed to engage today’s students in learning how to be information literate [47]. Librarians who support DVM students should (1) seek training opportunities in alternative modes of teaching and learning IL skills, and (2) partner closely with veterinary faculty and clinicians to provide students practice and feedback in information evaluation.

Our survey asked respondents to rate their confidence in and ease with reading scientific papers, but some studies have demonstrated that confidence is not a reliable gauge of skill or knowledge [48–50], and therefore, we cannot equate confidence with competence. In addition, we could not fully assess information-evaluation perceptions because 30.5% of respondents dropped out after completing part 1 of the survey (before reading the paper and/or completing the associated survey questions). Open-ended responses conveying discontent at being asked to read a paper for the survey seem to confirm previous observations about DVM student attitudes toward reading [13]. Respondents also invited us to consider other preferred ways of learning new information: “I prefer learning from experience and in hands on situations. There are a lot of factors in real life that scientific data can’t account for.”

Interpretation of our findings should consider the following limitations. Participation was only 6.5% across institutions, much lower than response rates to other surveys of DVM students across North America [51]. We do not know whether this was due to lack of incentive to participate, lack of time available for students to commit to taking it, lack of interest in the survey or subject matter, time of semester and other pressures or stressors, or other unknown factors. North American students are often surveyed, and this survey, with a linked scientific paper to be read, might have contributed to survey fatigue. The timing of the survey varied due to the variety of environments at each participating institution, including IRB and administrative approval processes, and due to the logistical barriers in administering the survey across multiple institutions, it was not possible to target nonparticipants for follow-up to encourage them to participate.

Our results might be skewed because a larger percentage of students with higher degrees responded to our survey. It is possible that DVM students with additional degrees were more likely to engage with a survey that required reading a scientific paper.

For the paper-reading task, our use of a paper on metacognition in student learning [33], rather than a veterinary topic, might have reduced respondent interest in completing that part of the survey. One student commented:

It really depends on the paper’s topic—majority of papers I read are in regards to current patients I have or animals that are in my care, so it is applicable and important to me. While the topic of this paper isn’t interesting to me, so I didn’t put much effort into trying to read it.

Many students perceived that they lacked the skills needed to evaluate scientific literature critically. Although reading an increased proportion of a paper positively correlated with increased confidence, many students based their opinions about a paper on the abstract, introduction, and conclusion. They also identified issues with the clinical application of knowledge gained from reading scientific literature, which is a key feature for practicing evidence-based medicine. To move beyond these issues, librarians must partner with veterinary faculty, clinical role models, curricular administrators, and educational researchers to understand how they can best contribute to cultivating information-literate practitioners in a time of competency-based curriculum reform. Further research with DVM students and practicing veterinarians is necessary to understand how they are learning, retaining, and using IL skills.

## DATA AVAILABILITY STATEMENT

Data associated with this article cannot be made available due to IRB restrictions.

## SUPPLEMENTAL FILES

Appendix Survey instrument

## 

**Erin R. B. Eldermire, MLS**, erb29@cornell.edu, http://orcid.org/0000-0001-5846-40, Head, Flower-Sprecher Veterinary Library, Cornell University Library, Veterinary College, Cornell University, Ithaca, NY

**Suzanne Fricke, DVM, MLIS, AHIP**, suzanne.fricke@wsu.edu, https://orcid.org/0000-0002-4412-9717, Animal Health Sciences Librarian, Animal Health Library, Washington State University, Pullman, WA

**Kristine M. Alpi, MLS, MPH, PhD, AHIP**, alpi@ohsu.edu, http://orcid.org/0000-0002-4521-3523, University Librarian, OHSU Library, Oregon Health & Science University, Portland, OR

**Emma Davies, BVSc, MSc**, ed445@cornell.edu, Associate Clinical Professor, Section of Neurology/Neurosurgery Section Chief, Cornell University, Ithaca, NY

**Andrea C. Kepsel, MLIS, AHIP**, akepsel@msu.edu, https://orcid.org/0000-0002-9651-6700, Health Sciences Educational Technology Librarian, University Libraries, Michigan State University, East Lansing, MI

**Hannah F. Norton, MSIS, AHIP**, nortonh@ufl.edu, http://orcid.org/0000-0001-8062-2763, Health Science Center Libraries, University of Florida, Gainesville, FL
