# Supplementary material for: Information seeking and evaluation: a multi-institutional survey of veterinary students
Source: J Med Libr Assoc. 2019 Oct 1;107(4):515–26. doi: 10.5195/jmla.2019.674 (PMC6774543; doi:10.5195/jmla.2019.674)
Supplement: Appendix [file jmla-107-515-s001.pdf]

JMLA Journal of the Medical Library Association

Supplemental content to
J Med Libr Assoc. Oct;107(4):dx.doi.org/10.5195/jmla.2019.674
www.jmla.mlanet.org
© Eldermire, Fricke, Alpi, Davies, Kepsel, Norton 2019

Information seeking and evaluation: a multi-institutional survey of veterinary students

Erin R. B. Eldermire, MLS; Suzanne Fricke, DVM, MLIS, AHIP; Kristine M. Alpi, MLS, MPH, PhD, AHIP; Emma Davies, BVSc, MSc; Andrea C. Kepsel; Hannah F. Norton, MSIS, AHIP

APPENDIX

Survey instrument

Dear veterinary student,

Recently, the American Veterinary Medical Association (AVMA) updated its accreditation standards. One of these updates states that, upon graduation, students must be "competent in retrieving, evaluating and efficiently applying information." You can read more about the changes on the AVMA website: Accreditation Policies and Procedures of the AVMA Council on Education (COE) and review the current standard 5 on "Information Resources" at COE Accreditation Policies and Procedures: Requirements.

In light of this update, little is known about how veterinary students retrieve, evaluate, and apply information, and universities are working to adapt to this updated standard.

To help us understand how you retrieve, evaluate, and apply information, **we thought we would ask you!** Please consider taking this survey to help shape what we know in this area.

This survey **requires very little text entry; most questions are click-to-answer**. However, we would be grateful if you would add your comments to the optional text entry boxes.

If you have any questions or concerns about this survey, please do **be in touch!**

Here are the details:

This survey should take approximately ten minutes to complete *plus* the time it will take to read a scientific paper. Questions included are about your general reading habits and perceptions of scientific literature.

Your participation is voluntary, and you may stop taking the survey, not complete the survey, or not even begin the survey, all without any affect on your class standing, grades, or relationships with your university or faculty. We will do all that we can to keep your responses confidential, but as with all things on the Internet, we cannot guarantee 100% confidentiality. With that in mind, we will neither collect nor disseminate any identifying information.

This survey was originally created in partnership with other institutions and is being distributed to veterinary students at your university. If you have any questions or comments, you can contact the Ethics Hotline at 866.293.3077 or www.ethicspoint.com. Also, please see specific institutional information below.

This survey has been certified as exempt by the university institutional review board (IRB) #506005657.

[INFORMATION OMITTED TO RETAIN ANONYMITY]

If you understand the above and agree to participate in this survey, please answer the questions below.

JMLA Journal of the Medical Library Association

Supplemental content to
J Med Libr Assoc. Oct;107(4):dx.doi.org/10.5195/jmla.2019.674
www.jmla.mlanet.org
© Eldermire, Fricke, Alpi, Davies, Kepsel, Norton 2019

Q1: At what institution are you enrolled as a veterinary student?

Q2: What year are you?

- o 1st year (class of 2020)
- o 2nd year (class of 2019)
- o 3rd year (class of 2018)
- o 4th year (class of 2017)
- o Other

Q3: With which gender do you identify?

- o Male
- o Female
- o Transgender
- o Other
- o Prefer not to answer

Q4: What is your prior academic experience? (choose all that apply)

- o High school or secondary school
- o Some college/university experience (no bachelor's degree)
- o Bachelor's degree
- o Professional degree (veterinary, law, medicine...)
- o Master's degree
- o Doctorate (PhD)
- o Other (please specify)

Q5: Which of the following *best* describes how often you read scientific papers (which includes journal articles, conference proceedings, scholarly opinion pieces, etc.).

- o Never [if Never selected, skip to Q13]
- o Yearly
- o Every six months
- o Monthly
- o Weekly
- o Daily
- o Not applicable

JMLA Journal of the Medical Library Association

SC
Supplemental content to
J Med Libr Assoc. Oct;107(4):dx.doi.org/10.5195/jmla.2019.674
www.jmla.mlanet.org
© Eldermire, Fricke, Alpi, Davies, Kepsel, Norton 2019

Q6: On average, how many hours do you spend reading scientific papers in the time frame that you indicated in the question immediately above?

	0	5	10	15	20	Not applicable ☐
hours						

Q7: When you read a scientific paper, approximately what proportion of the whole paper do you read?

	0	10	20	30	40	50	60	70	80	90	100
Proportion of paper											

Q8-Q12: Please indicate how strongly you agree or disagree with the following. I read scientific papers...

	Strongly disagree	Neutral	Strongly agree
...because it is required for classes that I take			
...for papers/projects that I create			
...to further my scientific knowledge/career			
...for fun			
Other (please specify)			

JMLA Journal of the Medical Library Association

SC
Supplemental content to
J Med Libr Assoc. Oct;107(4):dx.doi.org/10.5195/jmla.2019.674
www.jmla.mlanet.org
© Eldermire, Fricke, Alpi, Davies, Kepsel, Norton 2019

Q13: How do you discover scientific papers that you read? (choose all that apply)

- o From Blackboard, Moodle, Classlist, or similar
- o I subscribe to table of contents alerts
- o I browse specific journals
- o I read what is recommended for my journal club
- o I get recommendations from friends or colleagues
- o I find it via Facebook, Twitter, ResearchGate, or other social media sites
- o I search a specific database (e.g., PubMed, Google Scholar) or website (e.g., journal website, library website)
- o Other

Q14: Please indicate how strongly you agree or disagree with the following:

	Strongly disagree	Neutral	Strongly agree
I love to read scientific papers			

Q15: Please indicate how strongly you agree or disagree with the following:

	Strongly disagree	Neutral	Strongly agree
I find reading scientific papers very easy			

Q16: Why?

___

Q17: Please indicate how strongly you agree or disagree with the following:

	Strongly disagree	Neutral	Strongly agree
I feel confident about my ability to interpret data from scientific papers.			

Q18: Why?

___

JMLA Journal of the Medical Library Association

Supplemental content to
J Med Libr Assoc. Oct;107(4):dx.doi.org/10.5195/jmla.2019.674
www.jmla.mlanet.org
© Eldermire, Fricke, Alpi, Davies, Kepsel, Norton 2019

Thank you for your answers! Next, we will ask you to read a scientific paper.

Click on the link below to access and read a scientific paper. The paper will open in a new tab.

After you have finished reading the paper, please *return to this page*, and click on the NEXT PAGE button to complete this survey.

The role of deliberate practice in the acquisition of clinical skills

Q19: How long did you spend reading the paper?

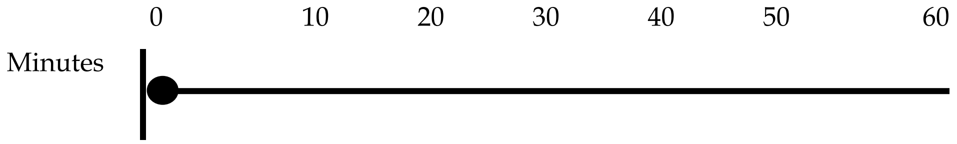

Q20: Did you read the entire paper?

- Yes [If Yes selected, skip to Q22]
- No [If No selected, continue to Q21]

Q21: What sections of the paper did you read? (choose all that apply)

- Title
- Authors and author affiliations
- Abstract
- Introduction/Background
- Methods
- Results
- Discussion
- Conclusions
- Additional material
- Acknowledgments
- References

Q22: Do the sections that you read for this paper generally reflect how you read scientific papers?

- Yes [If Yes selected, skip to Q24]
- No [If No selected, continue to Q23]

JMLA Journal of the Medical Library Association

Supplemental content to
J Med Libr Assoc. Oct;107(4):dx.doi.org/10.5195/jmla.2019.674
www.jmla.mlanet.org
© Eldermire, Fricke, Alpi, Davies, Kepsel, Norton 2019

Q23: What sections of the paper do you typically read? (choose all that apply)

- o Title
- o Authors and author affiliations
- o Abstract
- o Introduction/Background
- o Methods
- o Results
- o Discussion
- o Conclusions
- o Additional material
- o Acknowledgments
- o References

Q24–Q25: Please indicate how much you agree or disagree with the following statements:

	Strongly disagree	Neutral	Strongly agree
I found this paper very easy to read.			
I am confident that I understand the contents of this paper.			

Q26: After reading this paper, what are your take home points?

Q27: This paper presents information about the role of deliberate practice in the acquisition of clinical skills. If you were doing research on improving clinical skills, please indicate how much you agree/disagree with the following statement:

	Strongly disagree	Neutral	Strongly agree
I do not need any additional information on improving clinical skills.			

JMLA Journal of the Medical Library Association

Supplemental content to
J Med Libr Assoc. Oct;107(4):dx.doi.org/10.5195/jmla.2019.674
www.jmla.mlanet.org
© Eldermire, Fricke, Alpi, Davies, Kepsel, Norton 2019

Q28: Why?

Q29: Based on the results of this paper, I would...

- o Change my approach to learning clinical skills
- o NOT change my approach to learning clinical skills through adding deliberate practice
- o NOT change my approach to learning clinical skills because I already use deliberate practice

Q30: Why?

Your answers are valuable to us, and we appreciate the time that you have taken to fill out this survey. Please click the NEXT PAGE button to submit your survey. Thank you!
